# Breast MRI contrast enhancement kinetics of normal parenchyma correlate with presence of breast cancer

**DOI:** 10.1186/s13058-016-0734-0

**Published:** 2016-07-22

**Authors:** Shandong Wu, Wendie A. Berg, Margarita L. Zuley, Brenda F. Kurland, Rachel C. Jankowitz, Robert Nishikawa, David Gur, Jules H. Sumkin

**Affiliations:** 1 Department of Radiology, University of Pittsburgh, 4200 Fifth Ave, Pittsburgh, PA 15260 USA; 2 Magee-Womens Hospital of University of Pittsburgh Medical Center, 300 Halket St, Pittsburgh, PA 15213 USA; 3 University of Pittsburgh Cancer Institute, Department of Biostatistics, University of Pittsburgh, 4200 Fifth Ave, Pittsburgh, PA 15260 USA; 4 Department of Medicine, University of Pittsburgh, 4200 Fifth Ave, Pittsburgh, PA 15260 USA; 5 3362 Fifth Avenue, Pittsburgh, PA 15213 USA

**Keywords:** Breast cancer, Breast MRI, Normal parenchyma, Quantitative analysis, Contrast enhancement kinetics, Wash-in slope, Signal enhancement ratio

## Abstract

**Background:**

We investigated dynamic contrast-enhanced magnetic resonance imaging (DCE-MRI) contrast enhancement kinetic variables quantified from normal breast parenchyma for association with presence of breast cancer, in a case-control study.

**Methods:**

Under a Health Insurance Portability and Accountability Act compliant and Institutional Review Board-approved protocol, DCE-MRI scans of the contralateral breasts of 51 patients with cancer and 51 controls (matched by age and year of MRI) with biopsy-proven benign lesions were retrospectively analyzed. Applying fully automated computer algorithms on pre-contrast and multiple post-contrast MR sequences, two contrast enhancement kinetic variables, wash-in slope and signal enhancement ratio, were quantified from normal parenchyma of the contralateral breasts of both patients with cancer and controls. Conditional logistic regression was employed to assess association between these two measures and presence of breast cancer, with adjustment for other imaging factors including mammographic breast density and MRI background parenchymal enhancement (BPE). The area under the receiver operating characteristic curve (AUC) was used to assess the ability of the kinetic measures to distinguish patients with cancer from controls.

**Results:**

When both kinetic measures were included in conditional logistic regression analysis, the odds ratio for breast cancer was 1.7 (95 % CI 1.1, 2.8; *p* = 0.017) for wash-in slope variance and 3.5 (95 % CI 1.2, 9.9; *p* = 0.019) for signal enhancement ratio volume, respectively. These odds ratios were similar on respective univariate analysis, and remained significant after adjustment for menopausal status, family history, and mammographic density. While percent BPE was associated with an odds ratio of 3.1 (95 % CI 1.2, 7.9; *p* = 0.018), in multivariable analysis of the three measures, percent BPE was non-significant (*p* = 0.897) and the two kinetics measures remained significant. For the differentiation of patients with cancer and controls, the unadjusted AUC was 0.71 using a combination of the two measures, which significantly (*p* = 0.005) outperformed either measure alone (AUC = 0.65 for wash-in slope variance and 0.63 for signal enhancement ratio volume).

**Conclusions:**

Kinetic measures of wash-in slope and signal enhancement ratio quantified from normal parenchyma in DCE-MRI are jointly associated with presence of breast cancer, even after adjustment for mammographic density and BPE.

## Background

Breast magnetic resonance imaging (MRI) is recommended by the American Cancer Society as an adjunct to mammography for screening women who are at high risk of developing breast cancer [1]. Mammography is limited by low sensitivity and dense tissue can mask cancer detection [2]. In standard breast MRI protocols, dynamic contrast-enhanced MRI (DCE-MRI) using a gadolinium-based contrast agent provides a high intensity distinction between normal and diseased breast tissue, making it sensitive to breast tissue composition and microvascularity [3]. While mammographic breast density has been established as an independent risk factor [4–6], recent studies showed that MRI background parenchymal enhancement (BPE), visually assessed by the Breast Imaging-Reporting and Data System (BI-RADS) categories [7], is also associated with breast cancer risk [8, 9]. BPE represents the contrast enhancement of fibroglandular tissue in response to the MR contrast agent, and is typically assessed from a single sequence, namely the first post-contrast sequence, usually acquired at 90 seconds (k-space center time) after contrast agent administration [8, 9].

Breast DCE-MRI includes multiple (e.g., three) post-contrast sequences acquired at different time points after the injection of MR contrast agent [10]. The time-signal intensity curves of multiple post-contrast sequences reflect dynamic signal intensity changes induced by uptake of contrast agent over time, and can be described by contrast enhancement kinetics [11]. Typical kinetic curves are categorized at the voxel level as “persistent”, “plateau”, and “washout” [7]. Studies have shown that the kinetic curves of breast lesions have clinical diagnostic value for malignancy (wash-out curve) and benign (persistent curve) [11–13]. A set of common kinetic variables, such as wash-in slope, wash-out slope, time to peak, and peak enhancement, have been derived from the kinetic curves to specify characteristics of temporal contrast enhancement [14, 15], and have been associated with genetic estimates of breast cancer recurrence risk [16] and neoadjuvant chemotherapy response [17]. Other empirical variables of enhancement kinetics, such as the signal enhancement ratio (SER) computed based upon an early and a delayed post-contrast MR sequence, have also been derived as an imaging biomarker and shown useful for predicting benignity/malignancy [18] and breast tumor response to chemotherapy [10].

In addition to characterizing breast lesions, contrast enhancement kinetics computed from normal breast parenchyma (i.e., fibroglandular tissue) may capture certain physiologic/biologic characteristics of the breast as well. Contrast enhancement features involving both normal parenchyma and breast lesion have been shown indicative of breast tumor molecular subtypes [19]. A recent study showed a difference in the kinetic features of normal breast tissue between *BRCA1/2* mutation carriers and matched non-*BRCA* high-risk patients [20]. The kinetic variables derived from normal breast parenchyma are likely related to the risk of developing breast cancer. The purpose of this study was to investigate association between automatically computed quantitative contrast enhancement kinetics of normal parenchyma and presence of breast cancer in a case-control setting.

## Methods

### Study cohort

This retrospective study was compliant with the Health Insurance Portability and Accountability Act (HIPAA) and received Institutional Review Board (IRB) approval by the University of Pittsburgh, Human Research Protection Office (HRPO). Patient consent was waived. In a case-control setting this study included 102 women identified from an existing original research study. The original study had a separate IRB aimed at comparing the diagnostic performance of breast MRI, breast tomosynthesis, and computed tomography in women with known breast abnormalities, detected in a diagnostic setting by digital mammography, ultrasound, and/or clinical exam from January 2009 to December 2011 at our institution. Exclusion criteria were history of breast cancer, breast implants, lactating, benign breast surgery within one year, or ineligibility for breast MRI. A total of 154 women were recruited who had suspicious breast abnormalities and were rated as BI-RADS 4 or 5. These women consented to undergo bilateral breast MRI examinations before undergoing a percutaneous core and/or surgical biopsy. For premenopausal women, MRI was ideally scheduled the second week of the menstrual cycle but the actual date of MRI and date of onset of last menstrual period were recorded. Of the 154 women, pathological assessment confirmed 65 breast cancer cases and 89 benign lesions after MRI. In the present study MRI scans were assessed in 51 cases of unilateral cancer, excluding 14 cases of incomplete DCE subtraction sequences (missing due to informatics failure in archiving image scans). We implemented a case-control design with individual matching, controlling for unmeasured variability in factors associated with patients (by matching for age) and MRI techniques (by matching for year of MRI). Using a 1:1 ratio, 51 controls were selected from the 89 patients with unilateral biopsy-proven benign lesions, individually matched to patients with cancer by age (±3 years) and year of MRI (±1 year). Control status was affirmed by medical record review showing no diagnosis of breast cancer, with an average 3.7 years follow up (range 1.4–5.5 years). A total of 102 breast DCE-MRI scans were analyzed in this study.

### MRI protocols

MRI was performed at our institution using a standard and consistent clinical breast MRI protocol. Women were imaged in the prone position by a 1.5 T scanner (GE Signa EXCITE, GE Health, Nutley, NJ, USA) using a dedicated 7-channel surface array breast coil (InVivo, Gainesville, FL, USA). Imaging parameters were: matrix 512 × 512; slice thickness 2 mm; field of view 28–34 cm, flip angle 10°, repetition time (TR) 5.68 msec, echo time (TE) 2.736 msec. Bolus injection of the contrast agent, ProHance (Bracco Diagnostics, Princeton, NJ, USA), at 0.1 mmol/kg, 3 cc/sec was followed by a 20-cc saline flush. The first post-contrast sequence acquisition was centered at 90 seconds after contrast agent injection. A pre-contrast sequence and three sequential time point post-contrast sequences were acquired in the axial view for bilateral breasts, where each sequence took approximately 3 minutes to complete, depending on field of view sizes selected to cover the breasts. Three subtraction sequences (SUB1, SUB2, and SUB3) were generated by subtracting the pre-contrast sequence from each of the three post-contrast sequences, respectively, as part of routine post-processing with CADstream (Merge Healthcare Inc., Chicago, IL, USA).

### Kinetic variable quantification

**Fig. 1:**
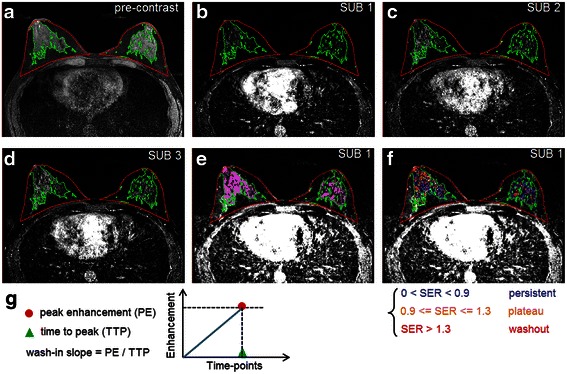
Automated measurement of contrast enhancement kinetic variables from normal parenchyma. **a** Automated segmentation of breasts (*red contour*) and fibroglandular tissue (*green contour*) from pre-contrast sequences. **b** The first time point subtraction (i.e., post-contrast–pre-contrast) sequence with superimposed segmentation. **c** The second time point subtraction sequence. **d** The third time point subtraction sequence. **e** Background parenchymal enhancement (BPE) (color-coded voxels in *purple*) quantified from the first time point subtraction (*SUB*) sequence. **f** Illustration of signal enhancement ratio (*SER*) quantification. The kinetics of each voxel was color-coded, based on the defined range of the voxel-wise SER values (see [10, 18]), as persistent (*blue*), plateau (*yellow*), or washout (*red*). SER volume is calculated as the total volume of voxels having SER ≥0.9 (i.e., those voxels that have either plateau or washout kinetics). **g** Calculation of wash-in slope from peak enhancement and time to peak, for each voxel of fibroglandular tissue; here peak enhancement and time to peak were identified voxel-wise through all three sequential subtraction sequences

Previously published automated computer algorithms [21–23] were employed to process breast DCE-MRI scans and quantify contrast kinetics (Fig. 1). Kinetic variables were generated breast-wise from the contralateral breasts of patients with cancer and controls. Standard kinetic variables of wash-in slope, wash-out lope, time to peak, peak enhancement, and SER [10, 14], were computed based on the pre-contrast and three post-contrast sequences (see the references for their mathematical definitions and illustrations). Details of the automated process are briefly described in the next two paragraphs.

First, the whole breast region was outlined from breast MR images [21] and the fibroglandular tissue contents were segmented over the whole breast region [22] (Fig. 1a). The segmentation masks of breast and fibroglandular tissue were translated to all three sequential subtraction sequences after rigid inter-sequence registration (Fig. 1b-d). For each voxel belonging to the fibroglandular tissue, voxel-wise values for each of the four kinetic variables (wash-in slope, wash-out slope, time to peak, and peak enhancement) were computed. Then the mean and variance of the voxel-wise kinetic values were calculated for each kinetic variable, generating eight kinetic measures in total. Preliminary univariate analyses of the eight kinetic measures showed that the variance of wash-in slope values (denoted by wash-in slope variance, Fig. 1g) is most likely to be associated with presence of breast cancer [24], and therefore, we chose to focus on examining the effect of wash-in slope variance.

Second, the SER was computed for each voxel over the fibroglandular tissue by:

$$ \mathrm{S}\mathrm{E}\mathrm{R}=\left(\mathrm{S}1\hbox{-} \mathrm{S}0\right)/\left(\mathrm{S}3\hbox{-} \mathrm{S}0\right)=\mathrm{S}\mathrm{U}\mathrm{B}1/\mathrm{S}\mathrm{U}\mathrm{B}3, $$

where S0, S1, and S3 are signal intensities of the same voxel in the pre-contrast, first post-contrast, and third post-contrast images, respectively [10]. Based on the range of voxel-wise SER values with respect to the persistent, plateau, and washout kinetics defined in previous breast MRI studies [18], and referring to a preliminary evaluation [25], we chose to test a volumetric measure (denoted by SER volume, unit: cm^3^) defined as the total volume of enhancing voxels having SER ≥0.9 (i.e., those voxels that have either plateau or washout kinetics; Fig. 1f).

For comparison purposes, MRI BPE was also quantified from DCE-MRI (Fig. 1e). BPE contains voxels that had at least 20 % enhancement on the signal intensities of the first post-contrast image relative to the corresponding pre-contrast image, referring to a previously published method [23]. The percentage of the volume of BPE over breast volume was derived and denoted as BPE%. Standard clinical assessment of mammographic density by BI-RADS breast density categories was retrieved from mammography reports of the digital mammograms acquired within 6 months prior to the analyzed MRI scans.

### Statistical analysis

The primary analysis compared the two kinetic measures (wash-in slope variance and SER volume) in the contralateral breasts of patients with cancer and controls. We first examined the odds ratios for breast cancer of the kinetic measures using univariate and multivariate conditional logistic regression, where the multivariate regression was controlled for three base factors: menopausal status (premenopausal or postmenopausal), family history of breast cancer, and BI-RADS-based mammographic density categories. Family history of breast cancer was encoded as binary (positive if at least one first, second, or third-degree family member was diagnosed with breast cancer). Ability of kinetic measures to distinguish patients with cancer from controls was also assessed using area under the receiver operating characteristic (ROC) curve (AUC) from unconditional logistic regression models. The likelihood ratio test was used to assess differences in the AUC. Effects of BPE% were also tested by univariate and multivariable logistic regression, and ROC analysis. All statistical tests were two-sided, with *p* < 0.05 considered statistically significant. Statistical analyses were performed using SAS software (version 9.3 SAS Institute, Cary, NC, USA).

## Results

### Patient and imaging characteristics

Table 1 summarizes characteristics of the study cohort and imaging measures. Based on mammography and/or ultrasound, 40 (78 %) of the 51 breasts studied in patients with cancer were classified as BI-RADS 5, and 47 (92 %) of the 51 breasts studied in controls with benign lesions were classified as BI-RADS 4. The four control group patients with a diagnostic BI-RADS 5 lesion and a benign result on percutaneous biopsy underwent confirmatory excisional biopsy to resolve the discrepancy between the radiological findings and the percutaneous biopsy pathological findings, and all four lesions were verified to be benign. The vast majority, i.e., 46 (90 %) of patients with cancer and of controls, were reported as BI-RADS 1 or 2 for the contralateral breast, and no malignancies were found at biopsy in those rated BI-RADS 4 for the contralateral breast (4 women in each of the cancer and control groups).

**Table 1:** Patient and imaging characteristics of the 102 patients including 51 breast cancer cases and 51 matched controls with biopsy-proven benign lesions

Patient/imaging characteristics		Cancer cases (*n* = 51)	Controls (*n* = 51)
Diagnostic BI-RADS findings in single-side breast on mammography and/or ultrasound			
Breast with lesion (cancer/benign)	BI-RADS 4	11 (22 %)	47 (92 %)
	BI-RADS 5	40 (78 %)	4 (8 %)
Contralateral (studied) breast	BI-RADS 1	27 (53 %)	21 (41 %)
	BI-RADS 2	19 (37 %)	25 (49 %)
	BI-RADS 3	1 (2 %)	1 (2 %)
	BI-RADS 4	4 (8 %)	4 (8 %)
	BI-RADS 5	0 (0 %)	0 (0 %)
Age, years, mean ± SD (range)		47.6 ± 7.4 (34–60)	47.1 ± 7.3 (31–60)
Menopausal status			
Premenopausal		28 (55 %)	30 (59 %)
Postmenopausal		23 (45 %)	21 (41 %)
Known pathogenic *BRCA1/2* mutation		2 (4 %)	0 (0 %)
History of prior breast cancer		0 (0 %)	0 (0 %)
Family history of breast cancer		26 (51 %)	31 (61 %)
Family history of ovarian cancer		3 (6 %)	0 (0 %)
Prior biopsy (>1 year prior to the studied biopsy)			
Atypia		1 (2 %)	0 (0 %)
Benign abnormality		9 (18 %)	6 (12 %)
Exogenous hormone use			
Hormone replacement therapy		7 (14 %)	5 (10 %)
Birth control pills		33 (65 %)	34 (67 %)
Tamoxifen		1 (2 %)	0 (0 %)
None		5 (10 %)	12 (24 %)
Oophorectomy		0 (0 %)	0 (0 %)
Mammographic density (visual BI-RADS density description)			
Fatty		2 (4 %)	1 (2 %)
Scattered fibroglandular density		14 (27 %)	13 (25 %)
Heterogeneously dense		32 (63 %)	33 (65 %)
Extremely dense		3 (6 %)	4 (8 %)
Background parenchymal enhancement, BPE%, mean ± SD (range)		44.6 ± 8.9 (20.9–62.0)	39.8 ± 11.4 (17.9–61.6)
Kinetics imaging measures, mean ± SD (range)			
Wash-in slope variance × 100 (unit)		2.90 ± 2.72 (0.48–15.42)	1.75 ± 1.26 (0.43–6.87)
Signal enhancement ratio volume, cm^3^		118.9 ± 85.6 (26.1–429.8)	80.4 ± 42.9 (21.1–194.5)

Data are numbers of subjects, with percentages in parentheses, unless stated otherwise. *BI-RADS* Breast Imaging-Reporting and Data System

The 51 cancer cases primarily involved invasive cancers, including 26 invasive ductal carcinoma (IDC), four invasive lobular carcinoma (ILC), one invasive mixed ductal-lobular carcinoma, 18 mixed IDC and ductal carcinoma in situ (DCIS), and two DCIS. Of the 49 invasive cancers, 37 were estrogen receptor (ER)-positive or progesterone receptor (PR)-positive, including 9 that were positive for human epidermal growth factor receptor 2 (HER2); 10 were ER-negative and PR-negative (including 9 that were triple negative); and 2 were missing receptor status. Tumor size measured 2 cm or less in 18 patients, 2–5 cm in 31 patients, and more than 5 cm in 2 patients, with a median size of 2.4 cm.

There were 24 matched pairs of premenopausal women and 17 matched pairs of postmenopausal women. Ten pairs were discordant for menopausal status because the primary matching was age (±3 years), not menopausal status. This resulted in four premenopausal cases having postmenopausal controls, and six postmenopausal cases having premenopausal controls. A total of 58 women from the full cohort were premenopausal, and of these, MRI was not performed in 15 women in each of the cancer and control groups during the second week of the menstrual cycle. Biopsies had been performed on either one or both breasts in those who had undergone prior biopsy. The rate of family history of breast cancer was similar in cases (51 %) and controls (61 %). Of note, six patients with cancer had additional risk factors (family history of ovarian cancer, *BRCA1/2* mutations, or prior atypia), compared to none in the control group. The use of an exogenous hormone was a mixture of current and past use, with the majority being past use (e.g., 76 % and 88 % of women in the cancer and control groups, respectively, had used birth control pills in the past). No patients in the study cohort had taken aromatase inhibitors.

Spearman’s correlation coefficients (SCC) showed moderate correlation with BPE% for each of wash-in slope variance (SCC = 0.56, *p* < 0.0001) and SER volume (SCC = 0.48, *p* < 0.0001), but these two kinetic measures were not correlated with each other (SCC = 0.17, *p* = 0.1). Wash-in slope variance (SCC = -0.18, *p* = 0.07) and SER volume (SCC = -0.21, *p* = 0.03) were both weakly negatively correlated with the established risk factor, mammographic density.

### Association between kinetic measures and presence of breast cancer

**Fig. 2:**
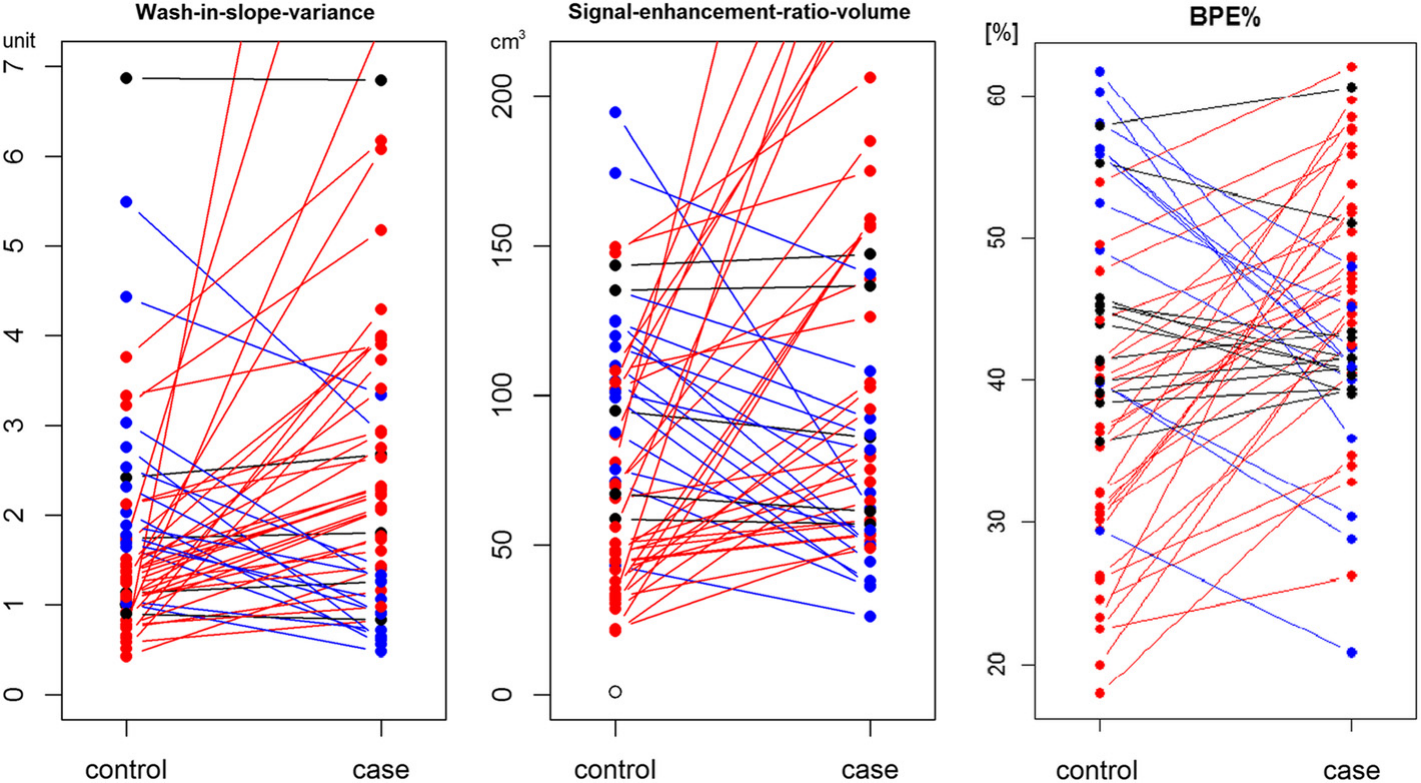
Kinetic variable and background parenchymal enhancement (*BPE*) comparisons between pairs of contralateral breasts in patients with cancer (*cases*) and controls matched by age and year of magnetic resonance imaging. *Dots* represent values of the measured kinetic variables or BPE%. *Line colors* encode differences between cases and controls as a percentage of the measure for controls. *Red lines* indicate pairs where the case value is >15 % greater than the control. *Blue lines* indicate pairs where the case value is >15 % less than the control. *Black lines* indicate pairs where the case value is within 15 % of the control value. This figure shows a trend of higher values measured in cancer cases compared to the matched controls for the two kinetic variables, wash-in slope variance (*left*) and signal enhancement ratio volume (*middle*), and the measure of BPE% (*right*)

**Fig. 3:**
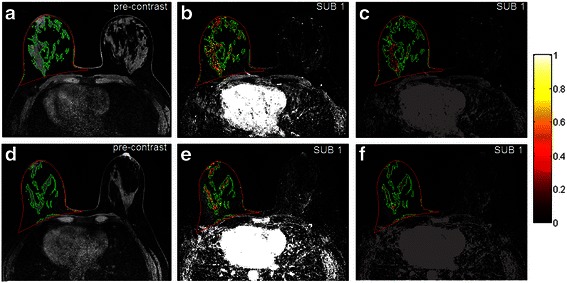
Selected representative slices of two magnetic resonance imaging (MRI) scans showing difference in the two kinetic variables between cancer cases and controls. *Row 1* is for a 58-year old postmenopausal woman with cancer (mixed invasive ductal carcinoma and ductal carcinoma in situ); *row 2* is for a 57-year old postmenopausal woman from the control group, who had a biopsy-proven benign lesion. In all plots, *red contours* outline the breast area and *green contours* outline the fibroglandular tissue. *Left column* (**a**, **d**) fibroglandular tissue segmentation. *Middle column* (**b**, **e**) color-coded voxels with a signal enhancement ratio (SER) value > =0.9 (*red* for washout kinetics having SER >1.3 and *yellow* for plateau kinetics having 0.9 < =SER < =1.3). These *colored voxels* on all slices were accumulated to compute SER volume (138.9 cm^3^ for the cancer case vs 102.0 cm^3^ for the controls). *Right column* (**c**, **f**) color-coded wash-in slope values. The *color bar* is for the two *rightmost* plots (**c**, **f**) only, denoting the range of the wash-in slope values. Wash-in slope variance (0.04 for the cancer case vs 0.01 for the control) was computed based on the wash-in slope values of color-coded voxels on all slices. *SUB* subtraction

In univariate analysis, ordinal mammographic density categories did not predict case/control status in this cohort (*p* > 0.45), while BPE% showed an association, with an odds ratio = 3.1 (95 % CI 1.2, 7.9), *p* = 0.018. Figure 2 shows comparisons of the MRI measures for the matched pairs. For both the two kinetic variables and BPE%, higher values occur more frequently in cases compared to matched controls. BPE% was at least 15 % higher in patients with cancer as compared to controls in over half of the matched pairs. For wash-in slope variance, 33 (65 %), 13 (25 %), and 5 (10 %) patients with cancer had values that were >15 % greater than, >15 % less than, and within 15 % of the corresponding values in the matched controls, respectively. For SER volume, 31 (61 %), 15 (29 %), and 5 (10 %) patients with cancer cases had values that were >15 % greater than, >15 % less than, and within 15 % of the corresponding values in matched controls, respectively. An example is shown in Fig. 3 for selected representative slices of two MRI scans illustrating differences in the two kinetic variables between cancer cases and controls.

Table 2 shows the results of univariate and multivariate conditional logistic regression analyses of the association between case-control status and the two kinetic measures and BPE%. The odds of malignancy were 1.7 times greater for each unit increase in wash-in slope variance (95 % CI 1.1, 2.7), and 3.1 times greater for each 100 cm^3^ increase SER volume (95 % CI 1.3, 7.5). These odds ratios remained very similar in models including both of the kinetic measures, even after adjustment for additional base risk factors (menopausal status, family history of breast cancer, and ordinal mammographic density) or the measure of BPE%. While univariate analysis showed a significant association (*p* = 0.018) between BPE% and cancer-control status, we noted that BPE% became non-significant (*p* = 0.897), and the two kinetics measures remained significant, when the three measures were jointly included in multivariate analysis. In addition, when excluding the six pairs in whom the patients with cancer (cases) had additional risk factors as described above, wash-in slope variance maintained a very similar association (*p* < 0.05) but association of SER became marginal (*p* > 0.056), after adjusting for the base factors or BPE%. The same phenomena for the two variables were observed when the 10 pairs mismatched for menopausal status were excluded from analysis.

**Table 2:** Odds ratios for breast cancer computed by univariate and multivariate conditional logistic regression analyses on the contralateral breasts of patients with cancer and controls (n = 102, 51 women with a cancer diagnosis and 51 controls with benign biopsy, matched by age and year of magnetic resonance imaging)

Conditional logistic regression models	Variables included in conditional logistic regression analyses		
	Wash-in slope variance (WISV) (unit)	Signal enhancement ratio volume (SERV) (cm^3^)	BPE% (%)
	OR (95 % CI); *p* value	OR (95 % CI); *p* value	OR (95 % CI); *p* value
WISV univariate	1.7 (1.1, 2.7); *p* = 0.014	-	-
SERV univariate	-	3.1 (1.3, 7.5); *p* = 0.014	-
WISV + SERV	1.7 (1.1, 2.8); *p* = 0.017	3.5 (1.2, 9.9); *p* = 0.019	-
Base factors + WISV + SERV^a^	1.8 (1.1, 2.9); *p* = 0.020	3.7 (1.2, 11.2); p = 0.020	-
BPE% univariate	-	-	3.1 (1.2, 7.9); *p* = 0.018
WISV + SERV + BPE%	1.7 (1.1, 2.8); *p* = 0.024	3.4 (1.1, 10.6); *p* = 0.038	1.1 (0.3, 3.8); *p* = 0.897

Odds ratio (OR) for WISV is per 0.01-unit difference. OR for SERV is per 100-cm^3^ difference. OR for percentage background parenchymal enhancement relative to breast volume (BPE%) is per 20 % point difference. ^a^Base factors = menopausal status (premenopausal vs postmenopausal), family history of breast cancer (yes/no, first to third degree family member), and Breast Imaging-Reporting and Data System (BI-RADS)-based mammographic density categories

### Preliminary performance of kinetic variables in classification

Unconditional logistic regression models were fitted to estimate the AUC for kinetic variables and BPE% as breast cancer screening classifiers (Table 3). Prediction of malignancy was significantly superior when both wash-in slope variance and SER volume were included in the regression model, compared to including either measure alone (both *p* = 0.005). The unadjusted (i.e., no cross-validation) and leave-one-out cross-validated AUC for a model with both variables as predictors was 0.71 and 0.68, respectively. When BPE% was added to the combination of the two kinetic variables, the unadjusted AUC was only marginally greater (AUC = 0.72) than without BPE% (AUC = 0.71), *p* = 0.775.

**Table 3:** Area under the receiver operating characteristic curve (AUC) for differentiation between patients with cancer and controls, analyzed by unconditional logistic regression models

Model (M)	Variables in unconditional logistic regression models	Unadjusted AUC (95 % CI)	Leave-one-out cross-validated AUC (95 % CI)	*P* value (likelihood ratio test)
M1	Wash-in slope variance (WISV) univariate	0.65 (0.54, 0.76)	0.61 (0.49, 0.72)	-
M2	Signal enhancement ratio volume (SERV) univariate	0.63 (0.52, 0.74)	0.59 (0.48, 0.70)	-
M3	WISV + SERV	0.71 (0.61, 0.81)	0.68 (0.58, 0.79)	M3 vs M1: 0.005
				M3 vs M2: 0.005
M4	BPE% univariate	0.64 (0.53, 0.75)	0.60 (0.49, 0.72)	-
M5	WISV + SERV + BPE%	0.72 (0.62, 0.82)	0.66 (0.56, 0.77)	M5 vs M3: 0.775
				M5 vs M4: 0.004

*BPE%* percentage of background parenchymal enhancement volume relative to breast volume

## Discussion

In this study, DCE-MRI contrast enhancement kinetic variables quantified from normal breast parenchyma were investigated in a case-control setting and were found to be associated with presence of breast cancer. Essentially, the measure of wash-in slope variance captures a form of voxel-wise heterogeneity of contrast uptake/enhancement in normal parenchyma, and the measure of signal enhancement ratio volume reflects the absolute amount of breast parenchyma that has a mixture of voxels with washout or plateau kinetics. To the best of our knowledge, this is the first study that quantitatively assessed MRI contrast enhancement kinetics of normal parenchyma for studying associations with the presence of breast cancer.

We showed that these two kinetics measures were jointly associated with the presence of breast cancer, indicating that they may convey complementary breast-cancer-related information. The associations were maintained even after adjusting for mammographic density and BPE%. BPE has been previously shown to be associated with breast cancer risk [8, 9] and it was also associated with the presence of breast cancer in this study. Our results suggest that the identified association between the presence of breast cancer and the two kinetic measures may extend to prediction of breast cancer risk.

Breast cancer is a biologically heterogeneous disease [26]. While the BI-RADS-category-based BPE assessment [8, 9] and the quantitative BPE% in this work characterize primarily the "amount" information on enhanced background parenchyma, the kinetic variables reflect some information on the voxel-wise heterogeneity in the abnormal contrast enhancement occurring in breast parenchyma that are still normal. The abnormal enhancement/vascularity characteristics may represent certain biological progression of potential disease development. Thus, compared to BPE, the kinetic variables are expected to capture some more relevant breast tissue profiles in relation to breast cancer risk. While both kinetic variables were correlated with BPE%, they remained as independent predictors of the presence of breast cancer, even after adjusting for BPE% in multivariate logistic regression analyses. However, future larger risk-assessment studies are warranted to further evaluate the value of DCE-MRI kinetics in prediction of breast cancer risk. While the kinetics of lesions have been used in the clinic for breast cancer diagnosis [11], the tested kinetic variables derived from normal parenchyma are expected to contribute to the estimation of breast cancer risk in the context of breast MRI screening. In this study, the MRI scans examined for the contralateral breasts of the patients with cancer were those obtained in patients with a current cancer diagnosis. It may be that contralateral breast kinetics and BPE% measured on women with known cancer in the other breast were actually elevated secondarily to the presence of the cancer. This case-control study implicates contralateral breast kinetic variables as markers of the presence of cancer, and we hypothesize that these may predict the development of breast cancer as well. To test this hypothesis, we plan to perform a follow up study using normal MRI scans acquired prior to diagnosis of cancer to further investigate the association between DCE-MRI kinetic variables and breast cancer risk.

In the context of breast cancer screening, existing risk models underestimate observed rates of breast cancer at the population level and are only moderate accurate at the individual level [27, 28]; for example, the AUC of the Gail, Claus, and Tyrer-Cuzick models is approximately 0.735, 0.716, and 0.762, respectively [29]. The ultimate goal of identifying new and significant imaging risk factors from quantitative breast MRI assessment would be to improve risk assessment accuracy and better guide individual decisions for more aggressive screening [30]. This study showed that the combination of two MRI kinetics measures alone yielded an AUC of 0.71, which is comparable to those of current risk models, although this is only a preliminary evaluation. We note that because the cases and controls in this study were matched by some risk factors (e.g., age) and not others (e.g., family history), our study precluded assessment of the incremental value of the kinetic variables over existing breast risk factors [31–33]. In future work, larger studies are warranted to fully examine the effect of incorporating the kinetic variables as potential risk biomarkers into current risk models.

Kinetic assessment of normal breast parenchyma has been shown to be related to the phase of the menstrual cycle in premenopausal women [34, 35]. About half of the 58 premenopausal women in our study had their MRI examinations outside the clinically recommended scanning window (i.e., second week of the menstrual cycle), 15 each for cases and controls. A recent study suggested that kinetic parameters of breast parenchyma were elevated when measured outside of the recommended interval, in patients with benign but not malignant lesions [35]. If that is the case, we would expect the measured DCE-MRI kinetics outside the second week in the 15 women with benign lesions in our control group to be higher than the actual levels. In the case-control analysis this would attenuate the effect of the kinetic measures between cancer and control groups. Despite this attenuation effect, we still found an association between breast DCE-MRI kinetics and presence of breast cancer.

There are several other limitations to our work. In this single-institution retrospective study, the sample size is relatively small and the MRI scans are consistent in the imaging protocol and parameters; thus generalizability of our results remains to be validated by a larger dataset, ideally in a multicenter study. Given the preliminary nature of this study mainly for generating hypotheses, multi-test correction was not applied and we believe the proof-of-concept results will be valuable in guiding study design and appropriate power calculations for larger studies. In addition, in the contralateral breasts of both patients with cancer and controls, there were some benign results (e.g., BI-RADS 2), which on one hand showed the robustness of the association of kinetics derived from a wider range of normal breast tissue, and on the other hand may have introduced bias by including benign findings in quantifying kinetics. Finally, kinetics of the normal parenchyma in the ipsilateral side of patients with breast cancer may merit investigation as well, but such an analysis remains for future work, as our current computer algorithms lack the function of separating normal parenchyma from breast tumor in cancer-affected breasts.

## Conclusions

In summary, breast DCE-MRI kinetic variables derived from normal breast parenchyma are associated with the presence of breast cancer, potentially independent of mammographic density and MRI BPE. In this study we reported fully automated computerized methods for quantifying kinetics. Such an approach is essential for conducting large quantitative studies and can accelerate the translational use of reproducible imaging biomarkers in the clinic. Our results support further investigation of quantitative DCE-MRI kinetics from normal parenchyma as a potential new risk biomarker aimed at ultimately improving prediction and management of breast cancer risk [36–38]

## Abbreviations

AUC, area under the curve; BI-RADS, Breast Imaging-Reporting and Data System; BPE, background parenchymal enhancement; *BRCA1/2*, Breast cancer susceptibility gene 1/2; DCE-MRI, dynamic contrast-enhanced magnetic resonance imaging; DCIS, ductal carcinoma in situ; ER, estrogen receptor; HER2, human epidermal growth factor receptor 2; HIPAA, health insurance portability and accountability act; IDC, invasive ductal carcinoma; ILC, invasive lobular carcinoma; IRB, institutional review board; MRI, magnetic resonance imaging; PR, progesterone receptor; ROC, receiver operating characteristic curve; SCC, spearman’s correlation coefficient; SER, signal enhancement ratio; SUB, subtraction
